# 

**Published:** 1885-03

**Authors:** 


					﻿i/Vhole Number, 303^
MARCH, 1885.
J/rrL-L, Number 3
THE
CHICAGO MEDICAL JOURNAL AND EXAMINER
EDITORS:
S. J. JONES, M.D., LL.D
W. W. JAGGARD, A M , M.D
HAROLD N. MOYER, M.D.
CHICAGO:
PUBLISHED BY THE CHICAGO MEDICAL PRESS ASSOCIATION,
240 Wabash Avenue.
CLARK & LONGLEY, PRINTERS. 183 AND 165 DEARBORN STREET, CHICAGO.
				

